# Bleaching Teeth

**Published:** 1895-04

**Authors:** D. D. Wiber

**Affiliations:** 717 11th st., N. W., Washington, D. C.


BLEACHING TEETH.—To be successful and improve the appearance of a darkened tooth, it is important to remember that the stain may be due to various chemical agents, which, in order to correct, each group will require a different bleaching material. That the bleaching may be permanent, it is necessary to be thorough, even if considerable tooth substance is sacrificed.

TREATMENT.—All decayed matter from crown-cavity and nerve canal should be removed, care being observed that the enamel remains uninjured, and as much dentine is left as possible in order to strengthen the tooth. The rubber dam should now be applied, embracing the tooth to be bleached and several adjoining ones. Rinse out the crown and canal with hot water, removing all decomposed and foreign substance. When the discoloration is of long standing the hue will be brown, bluish or black. In these cases pyrozone, or such agents as contain chlorine, should be resorted to. The Labarraque solution—chloride of sodium—which is highly commended, may be introduced on a pledget of cotton, and allowed to remain from thirty to sixty minutes, according to discoloration, the root canal being previously closed to prevent the caustic effect of this agent upon the soft parts. The chloride of lime is used in the same manner, using it repeatedly every fifteen minutes until desired effect is reached. Oxalic acid carefully applied yields good results.

Nitric acid will dissolve out amalgam residue, but it must be used with caution. This is manipulated by dipping the end of a finely pointed stick in the acid, and touching the discoloration every few moments; then syringe with water, completing the operation with some neutralizing solution like ammonia water to free it of acid. Cyanide of potassium, that deadly poison, will be useful for the same purpose, but it must be handled cautiously. Caustic pyrozone may be employed simply and advantageously. Apply dam as in previous cases, clean out cavity with care, wash with warm water and dry with hot air. To neutralize any acid reaction, flood floor and walls of cavity with cabonate of ammonia, dry, and apply, by means of bibulous paper on gold probe, the twenty-five per cent. caustic solution of pyrozone; this is evaporated; continue the application to the inner and outer surface of the tooth, evaporating each time, either with hot or cold blasts, until desired shade is established: irrigate profusely with water, dry out, and fill.

D. D. WIBER, D. D. S.
717 11th st., N. W., Washington, D. C.

